# Context-Dependent Clinical Narrative Signals of Renal Function Deterioration in Cross-Language Settings: Retrospective Multicohort Study

**DOI:** 10.2196/94249

**Published:** 2026-11-02

**Authors:** Andreas Antonakis, Eleni Drosataki, Eleftheria Kleio Dermitzaki, Christos Pleros, Dimitra Lygerou, Ariadni Androvitsanea, Nikolaos Papadakis, Konstantina Kydonaki, Alexandros Georgios Kavvadias, Myrto Konidaki, Georgios Aletras, Yannis Pantazis, Harikleia Gakiopoulou, Konstantinos Stylianou, Ioannis Petrakis

**Affiliations:** 1 Department of Nephrology, University Hospital of Heraklion, Voutes, Heraklion, 700 13, Greece; 2 School of Medicine, University of Crete, Voutes, Heraklion, Crete, Greece, 1 2813402183; 3 Department of Cardiology, Venizelio General Hospital of Heraklion, Heraklion, Crete, Greece; 4 FORTH Institute of Applied and Computational Mathematics, Heraklion, Crete, Greece; 5 Department of Pathology, National and Kapodistrian University of Athens, Athens, Attica, Greece

**Keywords:** natural language processing, kidney function deterioration, chronic kidney disease, renal function trajectory, electronic health record, clinical-status language analysis

## Abstract

**Background:**

Kidney Disease: Improving Global Outcomes (KDIGO) classifies chronic kidney disease (CKD) using categories based on both glomerular filtration rate and albuminuria. Structured measures remain essential but may not capture contextual clinical concerns documented in narrative records.

**Objective:**

This study examined whether text-derived clinical-status language features in clinician notes were associated with deterioration in renal function in independent Greek- and English-language clinical cohorts.

**Methods:**

We retrospectively analyzed a Greek general-nephrology cohort (314 patients; 10,306 estimated glomerular filtration rate [eGFR] measurements) and an independent English-language Medical Information Mart for Intensive Care IV (MIMIC-IV) replication cohort. The Greek primary analysis used a linear mixed effects model over a follow-up period of up to 1797 days. The MIMIC-IV primary analysis used a 90-day, patient-level fixed-horizon competing-outcome model (N=87,533). Separate analyses of Greek and MIMIC-IV data evaluated discrimination, calibration, and sensitivity to alternative feature specifications.

**Results:**

In the Greek cohort, a 1-SD higher negativity was associated with lower eGFR (*β*=−3.273 mL/min/1.73 m², 95% CI −5.049 to −1.498; *P*<.001) and a more negative time trajectory (time-by-score *β*=−1.324, 95% CI −2.553 to −0.096; *P*=.035). In MIMIC-IV, a 1-SD higher negative-vs-reassuring polarity contrast was associated with renal deterioration (relative risk ratio [RRR]=1.090, 95% CI 1.070‐1.110; *P*<.001). Within eGFR G categories, polarity-score areas under the curve (AUCs) were 0.506 to 0.552 using discharge eGFR and 0.518 to 0.534 using the worst observed eGFR. Discrimination for renal deterioration remained modest (one-vs-rest AUC=0.6134, 95% CI 0.6092‐0.6175), and adding both natural language processing (NLP) features to the clinical model increased AUC by only 0.0018 (95% CI 0.0010‐0.0025).

**Conclusions:**

Independently constructed clinical-language measures were associated with renal function deterioration across two languages and health care settings. The findings support cross-language replication of an association signal, not formal external validation, measurement equivalence, or clinically useful stand-alone prediction.

## Introduction

Chronic kidney disease (CKD) is a progressive condition and an increasing contributor to global morbidity and mortality [1]. Current clinical management uses the Kidney Disease: Improving Global Outcomes (KDIGO) glomerular filtration rate and albuminuria categories derived from structured laboratory data [2]. These measures are essential but may not capture all contextual clinical concerns documented during history taking and examination [3].

Clinical documentation converts findings from a patient’s history and examination into narrative text. The clinical history has long been recognized as a major contributor to diagnosis, yet much of this information is not represented in structured electronic health record (EHR) fields [3]. Consequently, clinically relevant narrative information may remain inaccessible to conventional risk-prediction models.

Natural language processing (NLP) enables systematic analysis of clinical narratives. In nephrology, patient-written session notes and emoticon sharing have been evaluated for remote monitoring of home hemodialysis (HD) in relation to technically troubled dialysis sessions [4].

This study examined whether cohort-specific, text-derived clinical status language measures were associated with renal function deterioration. The Greek cohort used a clinician-validated negativity score, whereas the Medical Information Mart for Intensive Care IV (MIMIC-IV) dataset used embedding-derived negativity and a negative-vs-reassuring polarity contrast (polarity). By comparing independent Greek- and English-language cohorts, we evaluated cross-language replication of a kidney outcome–related narrative signal.

## Methods

### Study Design and Data Sources

We performed a retrospective, multicohort analysis of EHR data from two distinct sources. The Greek source cohort comprised nephrology patients treated at Heraklion University Hospital, Crete, Greece (with data captured from 2020 2025). MIMIC-IV (which includes data captured from 2008 to 2022) provided an independent acute-care, English-language replication cohort [5]. Because the clinical populations, text pipelines, and outcome models differed, MIMIC-IV was not treated as a formal external validation dataset for the Greek model.

### Participants and Cohort Selection

The Greek clinical-text source cohort included 314 adults with at least two recorded renal function measurements and at least 90 days of observation: 170 with nondialysis CKD, 103 receiving maintenance HD (Table S1 in Multimedia Appendix 1), 22 with acute kidney disease in the context of CKD (AKD), and 19 undergoing kidney-transplant follow-up (NTx) (section S1 in Multimedia Appendix 1). No patients undergoing peritoneal dialysis were included. In the retained mixed effects analysis, dialysis was defined as the lowest renal function reference state. Recorded estimated glomerular filtration rate (eGFR) values in HD were not interpreted as native residual-kidney trajectories or as deterioration outcomes. Clinical-group coefficients were included only to adjust for the association between negativity and eGFR level and its change over time.

The MIMIC-IV text source included 331,793 NLP-scored admissions from 145,914 patients. The first eligible live discharge admission was selected for each patient. Complete-case denominators were 112,653 patients for 90-day mortality, 82,634 surviving patients for the renal deterioration model, and 87,533 patients for the fixed-horizon competing-outcome analysis. Cohort flow is depicted in detail in Figure S1 in Multimedia Appendix 1.

### Data Extraction and Preprocessing

Serum creatinine values were converted to eGFR using the Chronic Kidney Disease Epidemiology Collaboration (CKD-EPI) 2021 equation [6]. In MIMIC-IV, eGFR was categorized as G1 to G5. Albuminuria and evidence of chronicity were not consistently available, so these categories are eGFR G categories rather than complete two-dimensional KDIGO G-A strata. In the Greek model, baseline proteinuria was included as a separate continuous adjustment variable, but this did not constitute complete KDIGO G-A staging (section S2 in Multimedia Appendix 1). Greek trajectories were classified only after the text-derived score had been finalized.

### Feature Engineering

Clinical-status language features were constructed using cohort-specific pipelines. The Greek negativity used clinician-validated term frequency–inverse document frequency (TF-IDF) features. The English MIMIC-IV measures used normalized embeddings from the open source sentence-transformers/all-MiniLM-L6-v2 model (Sentence Transformers team) [7] and clinically curated adverse and reassuring anchor expressions. We focused on two primary measures: (1) negativity (neg_score; Greek cohort): the normalized TF-IDF weight assigned to clinician-retained adverse or worsening clinical-status expressions, and (2) polarity (MIMIC-IV): the negative-vs-reassuring semantic contrast derived from similarity to clinically curated adverse and reassuring anchor expressions.

TF-IDF weighting was used to quantify the relative importance of specific terms within the Greek narrative corpus [8] (Table 1; section S3 in Multimedia Appendix 1).

**Table 1.** Definitions and analytical roles of the text-derived clinical-status features.

Feature	Cohort	Definition	Analytical role
Negativity score	Greek	Sum of L2-normalized TF-IDF^a^ weights across the 21 clinician-retained category-term entries used to construct the Greek negativity score; standardized for modeling.	Primary clinical-language exposure in the longitudinal association model.
Negativity	MIMIC-IV^b^	Mean cosine similarity of each text chunk to 27 adverse anchors; summarized by admission maximum or 95th percentile and standardized.	Content feature; jointly modeled with the polarity contrast.
Reassuring-language similarity	MIMIC-IV	Mean cosine similarity of each chunk to 8 reassuring anchors.	Component used to construct the polarity contrast.
Negative-vs-reassuring polarity contrast	MIMIC-IV	Adverse-language similarity minus reassuring-language similarity; summarized by admission maximum or 95th percentile and standardized.	Primary MIMIC-IV renal association exposure.
Documentation volume	MIMIC-IV	Log-transformed number of text chunks, standardized in the primary model.	Utilization/acuity covariate; not a language-content measure.
eGFR^c^ G category	MIMIC-IV	CKD-EPI^d^ 2021 discharge eGFR categorized as G1 to G5.	Clinical adjustment; not complete KDIGO^e^ G-A staging.

a TF-IDF: term frequency–inverse document frequency.

b MIMIC-IV: Medical Information Mart for Intensive Care IV.

c eGFR: estimated glomerular filtration rate.

d CKD-EPI: Chronic Kidney Disease Epidemiology Collaboration.

e KDIGO: Kidney Disease: Improving Global Outcomes.

### Annotation-Based Content Validation

For the Greek pipeline, a native Greek-speaking nephrologist, blinded to the project, reviewed 86 candidate category-term entries in their original passages. Thirteen contextually irrelevant, ambiguous, anatomical, or treatment-related entries were removed (15.1%), leaving 73 validated entries. The Greek negativity score used in the reported analyses was constructed from 21 category-term entries initially classified as worsening/adverse and was defined as the sum of their L2-normalized TF-IDF weights. During subsequent review, 6 of these entries were reclassified and were therefore not included in the final 15-entry worsening subset presented in Multimedia Appendix 1; this reclassification did not alter the 21-entry score used in the reported analyses. One improvement entry was also subsequently reclassified and excluded from the final presented lexicon. For MIMIC-IV, investigators clinically curated 35 anchor expressions (27 adverse and 8 reassuring) and qualitatively reviewed the 3 highest-scoring adverse passages per processed admission. These procedures support content and face validity for clinical-status language, not psychometric calibration of emotional sentiment. Full lexicons, exclusions, reclassifications, anchor sets, and synthetic examples are provided in section S4 and Tables S2 to S5 in Multimedia Appendix 1.

### Statistical Analysis

The prespecified Greek primary analysis modeled 10,306 eGFR measurements from 314 patients over a follow-up period of up to 1797 days. The linear mixed effects model included patient-specific random intercepts and time slopes. Fixed effects comprised standardized time, standardized baseline negativity, and their interaction; age; gender; baseline eGFR; log-transformed baseline proteinuria; and clinical group, with dialysis as the reference state. Figure S2 in Multimedia Appendix 1 shows a directionally consistent association between higher adverse-language scores and renal deterioration across multiple sensitivity analyses. Clinical-group coefficients were adjustment terms and were not interpreted as novel subgroup findings. The analytic objective was to test whether adverse clinical-status language remained associated with eGFR level and change over time after adjustment for clinical factors. Figure 1 shows the fixed-effect predictions. A separate renal biopsy analysis used cubic B-splines restricted to 365 days (129 source patients and 6072 raw observations; complete-case model, 106 patients and 5118 observations) and is not represented in Figure 1. Additional details are provided in section S5 and Tables S6 to S8 in Multimedia Appendix 1.

To further investigate the temporal relationship between baseline negativity and renal function, we performed an exploratory longitudinal analysis including all 314 patients of the first Greek cohort. For each patient, the first creatinine measurement was retained, and to reduce the impact of times when a patient underwent frequent laboratory testing (such as during an episode of AKD), only a subset of the subsequent measurements was incorporated into the implemented analysis. This resulted in fewer creatinine observations being available for trajectory estimation than were present in the original dataset. In total, 7986 serum creatinine measurements were kept—the first measurement for each patient was considered as the observed baseline eGFR, while the remaining 7672 datapoints were retained at their actual observation times. Longitudinal eGFR trajectories were then examined using a linear mixed effects model incorporating a natural cubic spline for time with 3 degrees of freedom. The model included baseline negativity, expressed as a patient-level standardized score, its interactions with the spline terms, observed baseline eGFR, age, gender, and recorded baseline clinical class.

**Figure 1.**
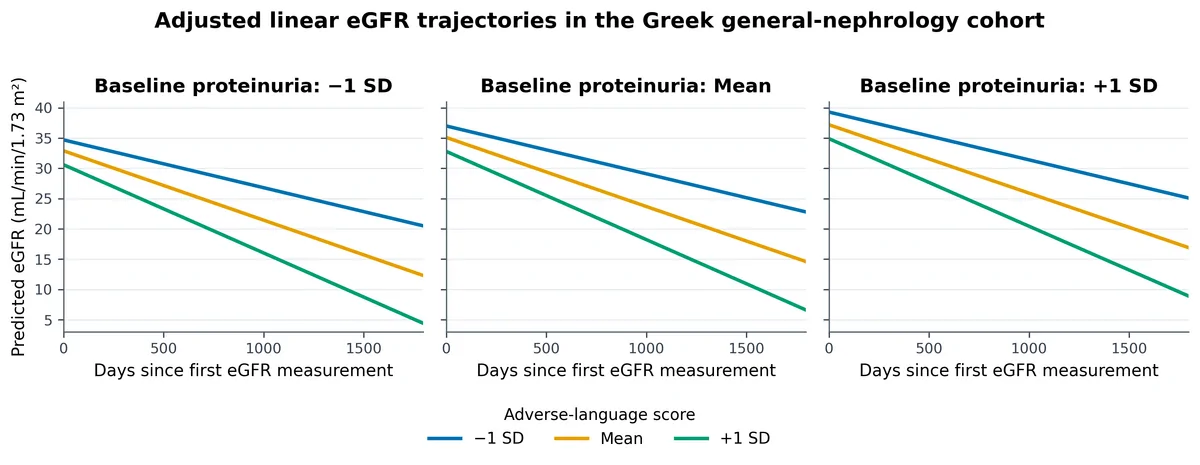
Model-predicted eGFR trajectories by negativity score and baseline proteinuria in the Greek general-nephrology cohort. Panels represent baseline proteinuria at −1, 0, and +1 SD; colored lines represent the negativity at −1, 0, and +1 SD. Other covariates were held at their mean or reference category. Lines show model-predicted mean glomerular filtration rate (eGFR); no confidence bands or error bars are displayed.

To evaluate whether baseline negativity was associated with subsequent changes in renal function after accounting for current renal status, we additionally performed a lagged interval analysis. For each pair of consecutive serum creatinine measurements, the outcome was defined as eGFR(t+Δt)−eGFR(t), where t denotes time. Models were conditioned on eGFR at the beginning of the interval, baseline clinical characteristics, elapsed follow-up time, prior laboratory measurement frequency, and the actual interval length. Spline-based terms were used to accommodate nonlinear changes over time. To reduce the influence of patients with more frequent laboratory testing, observations were weighted so that each patient contributed equal total weight.

Finally, the incremental predictive contribution of negativity was assessed using 5 repetitions of 5-fold cross-validation. Clinical-only and NLP-enhanced models were compared using root mean square error (RMSE), both for observed outcomes during the first year and across complete follow-up. CIs for differences in predictive performance were calculated by paired bootstrap resampling of the stored cross-validated predictions. This analysis represents an internal evaluation of the exported NLP scores and not an independent validation of the complete NLP processing pipeline.

The prespecified MIMIC-IV primary analysis used a patient-level fixed-horizon multinomial model with 3 mutually exclusive 90-day outcomes: alive without recorded renal deterioration, alive with renal deterioration, and death, with death prioritized (Figure S3 in Multimedia Appendix 1). Calibration of the primary MIMIC-IV models was assessed using 5-fold out-of-fold predictions (Figure S4 in Multimedia Appendix 1). Models were adjusted for age, sex, discharge eGFR G category, admission type, prior NLP-scored admissions, documentation volume, negativity, and the negative-vs-reassuring polarity contrast. For feature-specific clinical interpretation in the 82,634-patient survivor cohort, separate adjusted models containing polarity alone or negativity alone were reported first; the joint model was treated as a secondary conditional decomposition because the two text measures were correlated. Maximum feature aggregation was the primary analysis; 95th-percentile aggregation and inverse-probability weighting for renal outcome ascertainment were sensitivity analyses. Figures 2–4 show admission-level sensitivity analyses and are not the primary patient-level competing-outcome model. In a secondary admission-level analysis, the maximum polarity score was evaluated against the supplied 90-day renal deterioration composite within G1 to G5 categories defined by discharge eGFR and by the worst observed eGFR across the baseline-year median, inpatient nadir, and discharge value. Admission-level areas under the curve (AUCs) and 95% CIs were calculated using 1000 nonparametric bootstrap resamples in which patients, rather than admissions, were sampled as intact clusters. Additional details are provided in section S6 and Tables S9 to S16 in Multimedia Appendix 1.

**Figure 2.**
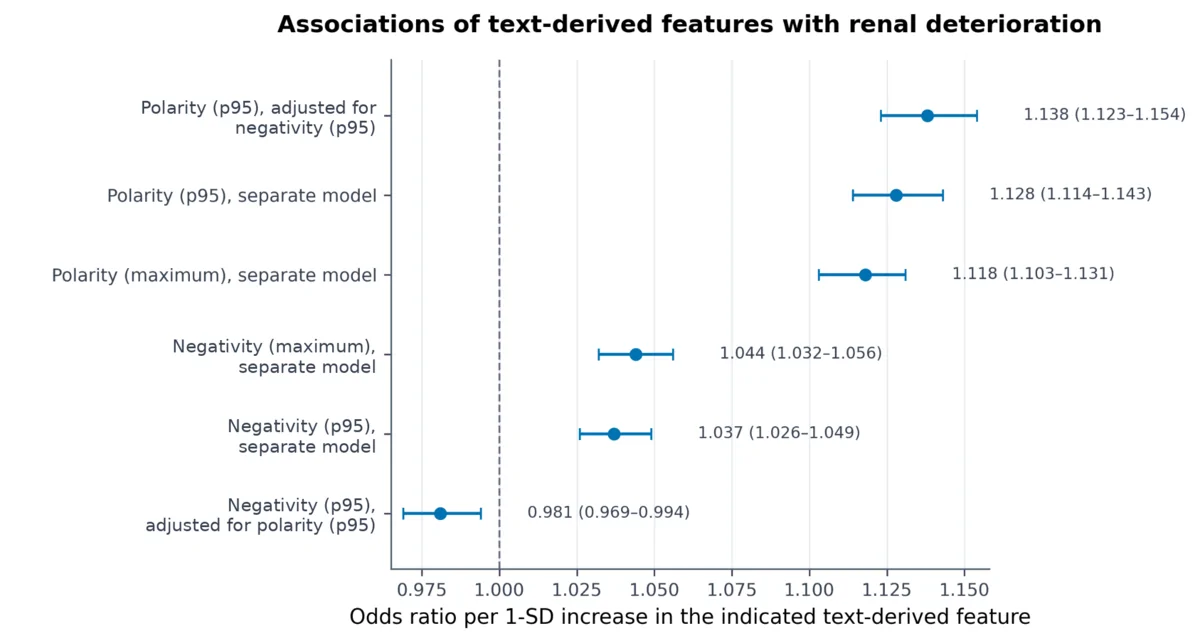
Admission-level associations of Medical Information Mart for Intensive Care IV (MIMIC-IV) text-derived features with the renal deterioration composite. Separate logistic models evaluated maximum and 95th-percentile (95p) negativity and negative-vs-reassuring polarity contrast; the combined 95th-percentile model included both features. Points are odds ratios per 1-SD increase and horizontal bars are model-based 95% CIs. The vertical dashed line denotes an odds ratio of 1.00.

**Figure 3.**
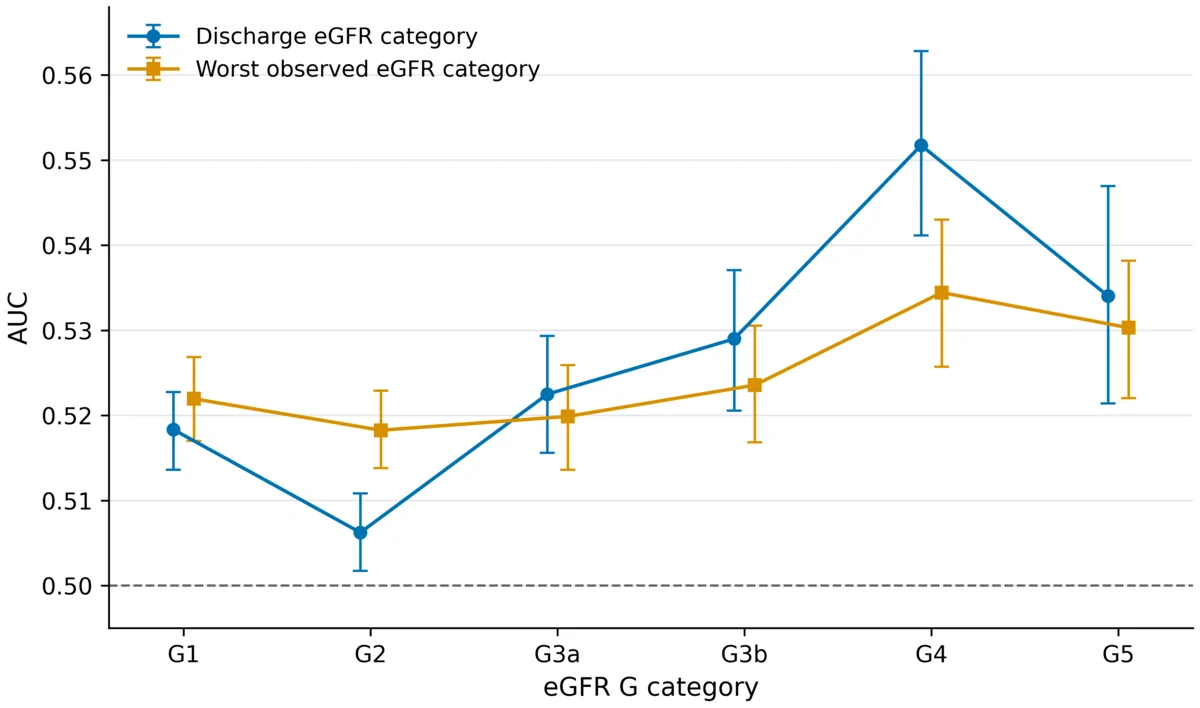
Medical Information Mart for Intensive Care IV (MIMIC-IV) discrimination of maximum polarity by estimated glomerular filtration rate (eGFR) G category. For G1, G2, G3a, G3b, G4, and G5, discharge-category sample sizes were 121,288, 78,875, 28,129, 19,986, 13,188, and 13,244 admissions; corresponding worst-eGFR sample sizes were 105,133, 97,678, 38,741, 31,776, 21,880, and 27,567. Error bars indicate percentile 95% CIs from 1000 patient-cluster bootstrap resamples. The dashed horizontal line denotes chance discrimination (area under the curve [AUC]=0.50). The G categories are based on eGFR alone and are not complete Kidney Disease: Improving Global Outcomes G–A strata.

**Figure 4.**
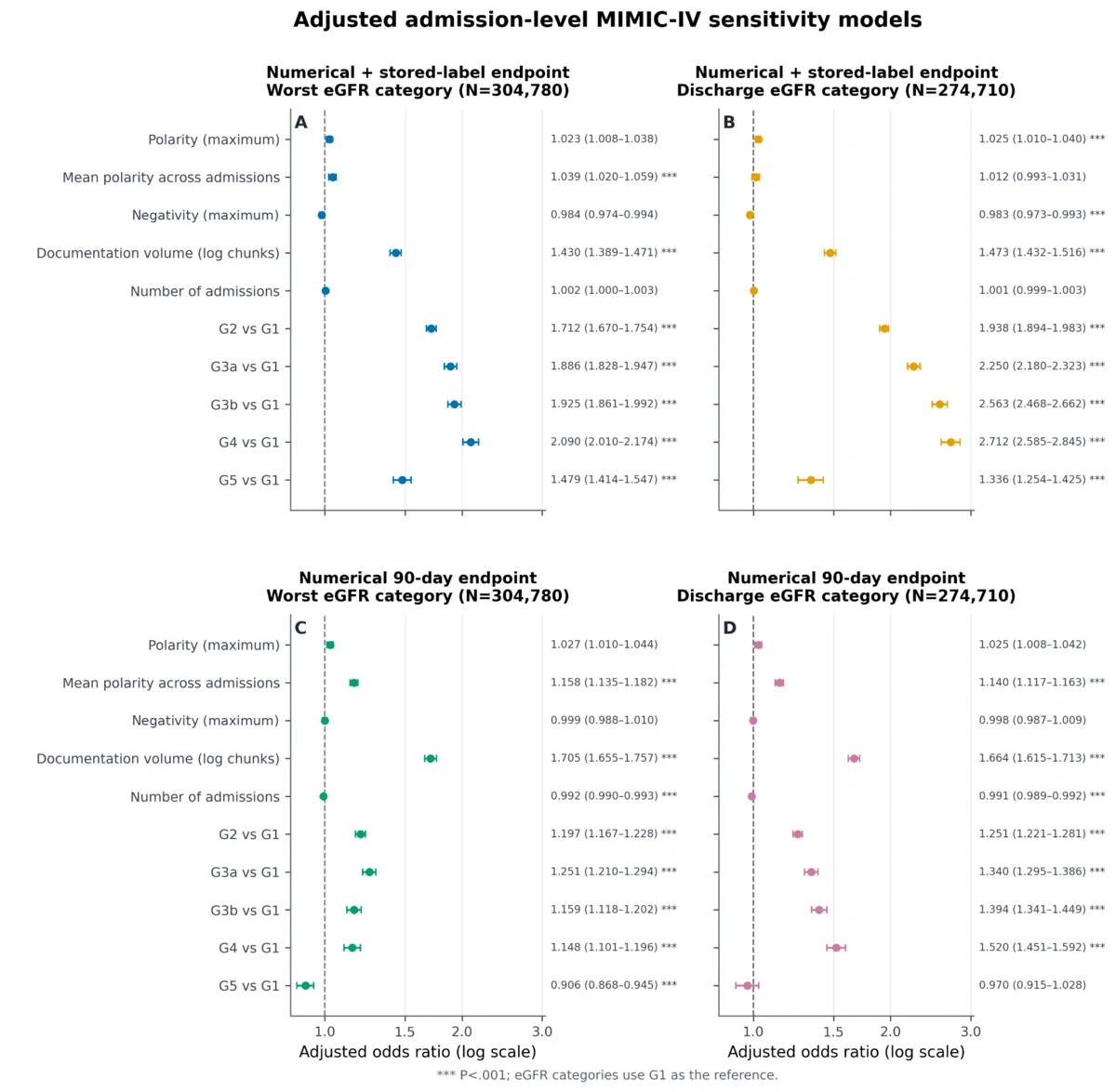
Adjusted admission-level Medical Information Mart for Intensive Care IV (MIMIC-IV) associations across renal end point and estimated glomerular filtration rate (eGFR) G category specifications. The four panels show multivariable logistic sensitivity analyses using the numerical 90-day renal deterioration composite alone or supplemented by a stored renal trajectory label, with renal status defined by discharge or worst eGFR. Worst-eGFR models included 304,780 complete admissions; discharge-eGFR models included 274,710. Each model included maximum polarity, patient-level mean maximum polarity, maximum negativity, log documentation volume, admission count, and eGFR G category (G1 reference). Points are adjusted odds ratios and horizontal bars are model-based 95% CIs. The dashed line denotes 1.00; asterisks denote *P*<.001. Documentation volume and admission count are utilization covariates.

A distinct Greek risk-model sensitivity analysis included 290 patients, of whom 136 had renal deterioration. The standard clinical model was a Gaussian linear mixed model for repeated eGFR, incorporating time, baseline age, sex, eGFR, log-transformed proteinuria, and clinical class, with time interactions for age, baseline eGFR, proteinuria, and clinical class and patient-specific random intercepts and slopes. Standard and NLP-enhanced AUCs were 0.724 and 0.714; the bootstrap AUC difference was −0.0109 (95% CI −0.0437 to 0.0210). This analysis is separate from the Greek longitudinal model and all MIMIC-IV analyses. All previous subcohorts used in various analyses are summarized in section S7 and Table S17 in Multimedia Appendix 1.

Covariates were clinically prespecified; no stepwise or outcome-driven variable selection was used. The Greek mixed model contained 10 nonintercept fixed-effect terms for 314 independent patient clusters (approximately 31 clusters per term) and converged. The standard-vs-NLP model comparison favored the NLP specification by Akaike information criterion (AIC; difference: −9.159) but not by Bayesian information criterion (BIC; difference: +5.322), so the findings are interpreted as adjusted associations rather than a fully validated predictive model. Primary analyses were specified separately for each cohort; no formal family-wise multiplicity adjustment was applied to secondary and sensitivity analyses, which were interpreted using effect sizes, CIs, and consistency. Analyses were conducted in Python version 3.14 using the pandas, patsy, and statsmodels packages (section S8 in Multimedia Appendix 1).

### Ethical Considerations

This study adhered to the Declaration of Helsinki and was approved by the institutional ethics committee of the University Hospital of Crete (approval number 230/07.03.2025). All data were deidentified prior to analysis. Informed consent was waived due to the use of anonymized retrospective data, in compliance with European Union General Data Protection Regulation standards (2016/679) (section S9 in Multimedia Appendix 1).

## Results

### Patient Characteristics

The Greek source cohort (N=314) comprised 170 patients with nondialysis CKD, of whom 103 received maintenance HD, 22 were diagnosed with AKD, and 19 were undergoing NTx follow-up. Mean follow-up was 610.23 (SD 436.96) days (maximum 1797 days). The higher descriptive mean eGFR in the NTx subgroup reflects expected functioning-graft status and was not treated as a study finding. Dialysis was the lowest renal function reference state, and all patient-group terms served only as adjustment covariates (Table 2). The MIMIC-IV text source comprised 331,793 admissions from 145,914 patients.

**Table 2.** Descriptive characteristics of the Greek clinical-text source cohort by clinical group. Elapsed time was calculated for each measurement relative to the patient’s first recorded measurement, assigned day 0. The reported range therefore includes baseline observations.

Variable	CKD^a^ (N=170)	HD^b^ (N=103)	AKD^c^ (N=22)	NTx^d^ (N=19)
Female sex, n (%)	76 (44.7)	49 (47.6)	11 (50.0)	12 (63.2)
Male sex, n (%)	94 (55.3)	54 (52.4)	11 (50.0)	7 (36.8)
Age (years), mean (SD)	68.5 (16.0)	66.8 (16.0)	64.0 (21.2)	52.7 (14.7)
Elapsed time (days) since baseline at recorded measurements (range)	484 (0‐1797)	519 (0‐1730)	533 (0‐1674)	699 (0‐1525)
Recorded eGFR^e^ (mL/min/1.73 m²), mean (SD)	38.0 (35.6)	13.9 (3.6)	37.2 (33.5)	46.5 (28.1)
Baseline proteinuria (mg/g creatinine), mean (SD)	1388.9 (2574.1)	2150.1 (5276.4)	898.6 (1161.0)	544.9 (559.8)

a CKD: nondialysis chronic kidney disease.

b HD: maintenance hemodialysis.

c AKD: acute kidney disease in the context of CKD.

d NTx: kidney-transplant follow-up.

e eGFR: estimated glomerular filtration rate.

### Primary Association Analyses

In the Greek primary analysis (314 patients; 10,306 eGFR measurements), a 1-SD higher negativity was associated with lower eGFR (*β*=−3.273 mL/min/1.73 m², 95% CI −5.049 to −1.498; *P*<.001) and a more negative time trajectory (time-by-score *β*=−1.324, 95% CI −2.553 to −0.096; *P*=.035) in each proteinuria class. These coefficients describe the adjusted clinical-language association and do not estimate effects of dialysis, transplantation, or AKD. Figure 1 displays fixed-effects predictions from the primary linear mixed effects model over the observed follow-up period.

In the separate Greek renal biopsy cohort, cubic B-spline models examined eGFR trajectories over 365 days. The source dataset included 129 patients and 6072 records; the complete-case analysis included 106 patients and 5118 records. After adjustment for baseline eGFR, age, sex, and proteinuria, negativity was associated with the modeled trajectory shape through its interaction with the time-spline terms (joint Wald *P*<.001). The proteinuria-by-time interaction was also significant (*P*<.001). Within the renal biopsy dataset, separate patient-level linear-slope metaregressions did not identify a significant negativity association after baseline adjustment. The spline findings and these linear-slope sensitivity results are summarized in Table 3. These secondary analyses are distinct from the primary 314-patient linear mixed effects analysis, which identified a significant time-by-negativity interaction and is illustrated in Figure 1.

**Table 3.** Results of the separate 365-day cubic B-spline analysis in the Greek renal biopsy cohort (129 source patients; complete-case run, 106 patients and 5118 observations). This analysis is distinct from the 314-patient linear mixed effects analysis displayed in Figure 1.

Analysis feature	Result	Interpretation
Baseline eGFR^a^ contrast	The high-negativity group started below the low-score group.	An early eGFR disadvantage persisted over follow-up.
Time-averaged eGFR contrast (high vs low negativity score)	Trajectory area/365=−4.41 mL/min/1.73 m² (95% CI −8.68 to −0.25).	Average predicted eGFR shortfall over 365 days; not a receiver operating characteristic area under the curve.
Cubic-spline time× negativity	Joint Wald *χ*² approximately 35‐107; *P*<.001 across specifications.	The language score was associated with nonlinear trajectory shape.
Cubic-spline time × proteinuria	Joint Wald test for the time × proteinuria interaction; *P*<.001. Proteinuria retained an independent association when modeled with the language score.	Proteinuria and clinical-status language provided complementary adjustment information.
Model-implied 365-day average contrasts	High vs low negativity approximately −5.4; high vs low proteinuria approximately −4.6 mL/min/1.73 m².	The predicted magnitudes were comparable.
Combined high language score and high proteinuria	Approximately −10 mL/min/1.73 m² vs the low-low group.	Predicted detriments were approximately additive.
Patient-level linear-slope metaregressions	No significant negativity association after baseline adjustment.	Simple linear slopes did not capture nonlinear or crossing patterns.

a eGFR: estimated glomerular filtration rate.

For clinically interpretable, feature-specific estimates in the 82,634-patient survivor cohort (MIMIC-IV), adjusted models containing one text feature at a time were examined prior to the secondary joint model. Polarity alone was associated with renal deterioration (odds ratio [OR]=1.080, 95% CI 1.061‐1.099), whereas negativity was not (OR=1.005, 95% CI 0.989‐1.021). In the joint model, polarity remained positively associated (OR=1.092, 95% CI 1.071‐1.112), and the negativity coefficient became weakly inverse (OR=0.973, 95% CI 0.956‐0.991), consistent with conditional suppression rather than protection (Table S15 in Multimedia Appendix 1).

In the MIMIC-IV fixed-horizon competing-outcome analysis (n=87,533; 57,813 alive without recorded deterioration, 24,821 with renal deterioration, and 4899 deaths within 90 days), a 1-SD higher negative-vs-reassuring polarity contrast was associated with renal deterioration (relative risk ratio [RRR]=1.090, 95% CI 1.070‐1.110; *P*<.001). Conditional on polarity and clinical covariates, negativity was weakly inverse for the renal category (RRR=0.973, 95% CI 0.956‐0.991; *P*=.003) but strongly positive for death (RRR=1.216, 95% CI 1.173‐1.261; *P*<.001). Given moderate correlation between the two content measures (Pearson *r*=0.468; Spearman ρ=0.450) and low maximum-feature variance inflation factors (polarity 1.384‐1.389; negativity 1.309‐1.315; Table S11 in Multimedia Appendix 1), the inverse relationship for renal deterioration is interpreted as conditional suppression, not a renoprotective association (Figures 2 and 4, Table 4).

**Table 4.** Primary adjusted Medical Information Mart for Intensive Care IV (MIMIC-IV) fixed-horizon competing-outcome model (N=87,533). Relative risk ratios (RRRs) are per 1-SD increase, with alive without recorded renal deterioration as the reference outcome. The two text-content measures were included simultaneously with age, sex, admission type, discharge estimated glomerular filtration rate (eGFR) G category, prior natural language processing–scored admissions, and documentation volume.

Predictor, per 1 SD	Renal deterioration, RRR (95% CI)	Death within 90 days, RRR (95% CI)
Polarity	1.090 (1.070‐1.110)	1.021 (0.982‐1.061)
Negativity	0.973 (0.956‐0.991)	1.216 (1.173‐1.261)
Documentation volume	0.988 (0.970‐1.006)	1.418 (1.367‐1.472)
Age	0.775 (0.761‐0.791)	2.550 (2.426‐2.681)

### Cross-Language Replication and Predictive Performance

The MIMIC-IV analysis constitutes an independent replication across a different language and clinical setting and of a severity-linked adverse-language signal associated with renal deterioration rather than validation or transport of the Greek TF-IDF model or replication of an equivalent linguistic construct. The exposure definitions, clinical populations, time horizons, and statistical models differed; consequently, the cohorts were analyzed separately and their coefficients were not pooled. Directional consistency was also observed in a harmonized binary sensitivity analysis (139 patients: Greek held-out score OR=1.48, 95% CI 1.03‐2.12; MIMIC-IV polarity OR=1.092, 95% CI 1.071‐1.112). Taken together, these findings suggest that adverse narrative content may capture a broader signal that is associated with renal outcomes across settings, but they do not establish cross-language equivalence of the underlying NLP constructs. Matching cohorts by sample size would mean discarding information without resolving these differences in constructs and settings. Polarity emerged as the strongest narrative predictor of disease severity among the evaluated language-derived features and appears to be the most promising candidate for predicting renal deterioration in future studies using comparable designs.

In the admission-level stage-specific sensitivity analysis, maximum-polarity AUCs using discharge eGFR categories were 0.518 (95% CI 0.514‐0.523) for G1, 0.506 (95% CI 0.502‐0.511) for G2, 0.522 (95% CI 0.516‐0.529) for G3a, 0.529 (95% CI 0.521‐0.537) for G3b, 0.552 (95% CI 0.541‐0.563) for G4, and 0.534 (95% CI 0.521‐0.547) for G5. Corresponding values using the worst observed eGFR were 0.522 (95% CI 0.517‐0.527), 0.518 (95% CI 0.514‐0.523), 0.520 (95% CI 0.514‐0.526), 0.524 (95% CI 0.517‐0.531), 0.534 (95% CI 0.526‐0.543), and 0.530 (95% CI 0.522‐0.538), respectively. All intervals were generated by patient-clustered bootstrap resampling. Although the estimates were consistently above 0.50, their magnitude remained near chance and does not support stand-alone individual-level discrimination (Table S16 in Multimedia Appendix 1).

Sensitivity analyses supported the MIMIC-IV polarity association (95th-percentile RRR=1.118, 95% CI 1.097‐1.139; inverse-probability-weighted RRR=1.074, 95% CI 1.053‐1.095). Prediction remained limited: the NLP-enhanced primary model had a one-vs-rest AUC of 0.6134 (95% CI 0.6092‐0.6175) for renal deterioration, and adding both NLP content features to the clinical base (CKD category) increased the AUC, which started at 0.6116 (95% CI 0.6074‐0.6159), by 0.0018 (95% CI 0.0010‐0.0025); reduced the multiclass Brier score by 0.00056 (95% CI 0.00037‐0.00074); and reduced log loss by 0.00120 (95% CI 0.00085‐0.00154). As expected from the present study design, these small increments do not establish clinical utility.

### Exploratory Longitudinal Analysis of NLP-Derived Negativity and eGFR Trajectories

In the exploratory mixed effects analysis of longitudinal eGFR trajectories, higher baseline negativity was not significantly associated with a different subsequent eGFR trajectory. At 365 days, the estimated eGFR difference associated with a 1-SD higher baseline negativity, conditional on the same observed baseline eGFR, was −1.41 mL/min/1.73 m² (95% bootstrap interval −5.05 to 2.07). The joint test of the negativity-by-time terms was not significant (*P*=.332). Furthermore, a maximum-likelihood comparison of models fitted to identical observations did not support improved model fit after inclusion of the 4 negativity-related terms, with ΔBIC=+28.30.

Consistent with these findings, the lagged interval analysis did not demonstrate evidence that higher baseline negativity was associated with greater subsequent eGFR decline after accounting for current eGFR and other covariates. Across 7672 consecutive serum-creatinine intervals, the median interval was 1.02 days (IQR 0.97‐1.94), and the mean was 24.98 (SD 112.53) days, reflecting a strongly right-skewed distribution. The model incorporated actual interval lengths through spline increments. The estimated difference associated with a 1-SD higher baseline negativity score was 0.48 mL/min/1.73 m² (95% patient-bootstrap interval −1.79 to 2.50), averaged across the observed intervals with equal total patient weighting. This estimate represents an interval-change contrast, not a daily rate, annualized slope, or fixed 365-day change.

Cross-validated prediction analyses similarly showed no meaningful improvement in first-year predictive performance after the addition of the negativity score. Among 4887 observed first-year outcomes from 289 patients, RMSE was 29.277 mL/min/1.73 m² for the clinical-only model and 29.281 mL/min/1.73 m² for the NLP-enhanced model, corresponding to a difference of 0.003 mL/min/1.73 m² (95% conditional paired-bootstrap interval, −0.160 to 0.199). In the exploratory natural-cubic-spline prediction analysis, complete follow-up RMSE was 31.889 mL/min/1.73 m² for the clinical-only model and 32.129 mL/min/1.73 m² for the NLP-enhanced model. The difference of 0.240 mL/min/1.73 m² (conditional paired-bootstrap 95% CI 0.004‐0.499) indicated a small increase in prediction error and slightly worse predictive performance after adding the negativity terms in this internal validation.

These findings complement the previously observed model-dependent interaction and support interpretation of adverse linguistic features primarily as potential markers of clinical state rather than established predictors of the subsequent rate of renal deterioration.

### Risk-Model Sensitivity Analysis

In the separate 290-patient Greek risk-model sensitivity analysis, the standard model had an AUC of 0.724, and the NLP-enhanced model had an AUC of 0.714; the patient-level bootstrap difference was −0.0109 (95% CI −0.0437 to 0.0210; Table S8 in Multimedia Appendix 1). Threshold-specific incremental net-benefit differences (NLP-enhanced minus standard model) were 0 at thresholds 0.05, 0.10, and 0.15; 0.0009 at 0.20; 0.0023 at 0.25; 0.0212 at 0.30; −0.0098 at 0.35; 0.0034 at 0.40; 0.0050 at 0.45; and 0.0172 at 0.50 (Figure 5 and Table S14 in Multimedia Appendix 1).

A summary of all subcohorts mentioned in this manuscript is provided in Table S17 in Multimedia Appendix 1.

**Figure 5.**
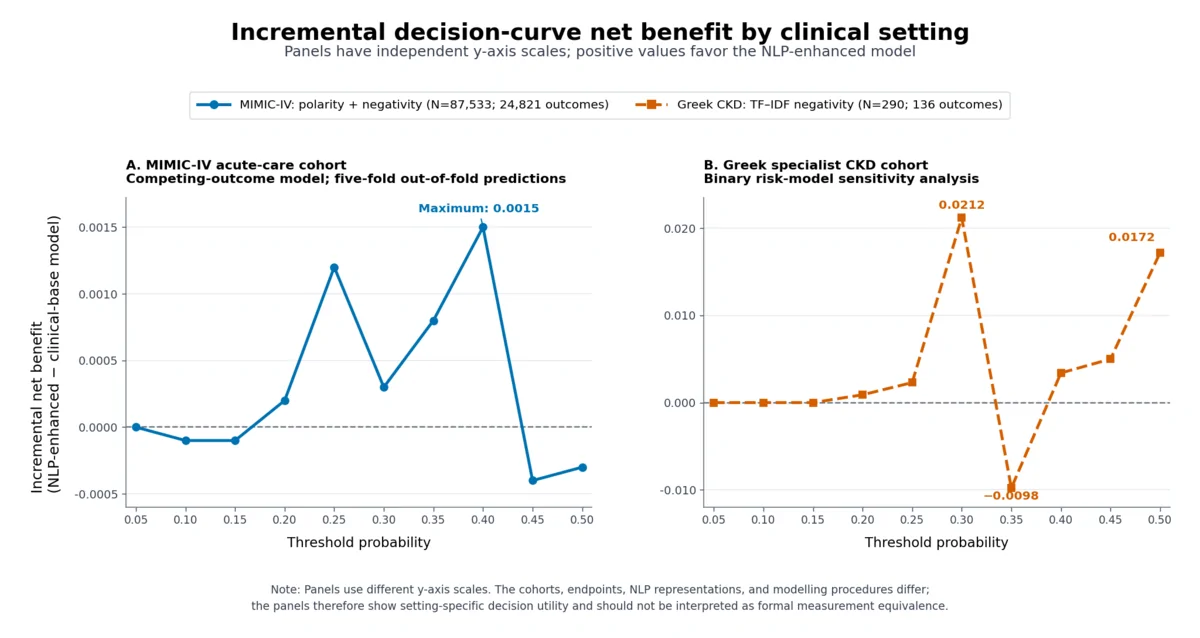
Incremental decision-curve net benefit across clinical settings. The curves show the difference in net benefit between the natural language processing (NLP)–enhanced and clinical-base models across threshold probabilities of 0.05 to 0.50; positive values favor the NLP-enhanced specification. The Medical Information Mart for Intensive Care IV (MIMIC-IV) curve (blue solid line; circles) uses 5-fold out-of-fold renal deterioration probabilities from the patient-level competing-outcome model (N=87,533; 24,821 renal deterioration outcomes), with maximum polarity and negativity added to the clinical base model. The Greek chronic kidney disease (CKD) curve (orange dashed line; squares) represents the separate binary risk-model sensitivity cohort (N=290; 136 deteriorations), with the clinician-validated term frequency–inverse document frequency (TF–IDF) negativity score added to the standard clinical model. The panels use independent y-axis scales. Each panel evaluates decision curves within its own cohort and end point. Differences in eligibility, clinical setting, event definition, observation window, linguistic features, and model evaluation preclude direct numerical comparison between panels.

## Discussion

### Principal Findings

While proteinuria is a key determinant in risk stratification, its prognostic value varies within CKD categories and is influenced by the broader disease context. Contemporary guidance emphasizes serial assessment of eGFR and albuminuria rather than reliance on a single measurement [2]. Viewing renal decline longitudinally can capture clinically important progression that may be obscured by static staging.

Clinical narratives contained information indicating deterioration in renal function in both cohorts. The Greek negativity score was associated with longitudinal eGFR level and trajectory, whereas the MIMIC-IV negative-vs-reassuring polarity contrast was associated with short-term renal deterioration. Because the NLP pipelines, outcomes, and care settings differed, these findings support cross-language convergence of a kidney-related narrative signal rather than formal measurement invariance or language-driven differences.

The stage-specific analysis refines this interpretation. The polarity score remained weakly discriminatory within every broad discharge and worst-observed eGFR G category, and each patient-clustered 95% CI lay above 0.50. Thus, the association in the notes was not explained solely by differences between eGFR G categories. Nevertheless, AUCs of 0.506 to 0.552 and 0.518 to 0.534 represent only a small ranking signal. The large MIMIC-IV cohort independently confirmed this association with high statistical precision (owing to the large sample size), showing that routine English-language clinical notes carry a detectable renal outcome signal. However, this study primarily establishes the existence and reproducibility of this signal across different settings, rather than positioning the current feature as an individual risk classification tool. This distinction highlights the importance of evaluating clinical-language measures within their specific corpus, care setting, and end point.

We demonstrated that narrative polarity is associated with renal outcomes, indicating that unstructured clinical text contains prognostically relevant information beyond routinely available structured variables. Although we did not demonstrate that incorporating this narrative information significantly improves the performance of a prediction model, our findings support the potential value of such approaches and warrant further assessment in prospective studies specifically designed to evaluate the incremental predictive utility of narrative-derived features.

### Comparison With Existing Literature

Prior work has established the value of renal NLP but addressed different questions. Perotte et al [9] combined topics derived from primary care clinical notes with longitudinal laboratory data to predict CKD progression, showing that incorporating clinical documentation to existing laboratory-based prediction models improved prediction of CKD progression from stage III to stage IV. Sun et al [10] integrated ICU notes with structured physiological measurements to predict incident acute kidney injury, slightly improving the prediction accuracy when notes were added to the model. Zha et al [11] incorporated nursing-note sentiment scores into mortality models for patients with severe acute kidney injury receiving continuous renal replacement therapy, demonstrating that sentiment information provided additional prognostic value for mortality. Garcia-Lopez et al [12] accurately classified kidney-allograft loss from Spanish-language electronic medical records. These studies were all single-language prediction or classification studies.

To our knowledge, no previous study has independently constructed clinically reviewed language measures in Greek and English and demonstrated their convergent association with objective renal function outcomes and measured renal trajectories across specialist nephrology and acute hospital settings. The present study therefore demonstrates that routine clinical narratives contain a clinically relevant signal of renal function deterioration across languages and care settings, supporting the future evaluation of narrative-derived features as predictors that may enhance the accuracy of renal deterioration models. Because the two pipelines were developed independently, this contribution represents cross-language conceptual replication of the renal status construct rather than external validation of a single transferable algorithm.

### Limitations

This study is retrospective and may be subject to confounding due to treatment changes, acute illness, socioeconomic factors, and documentation practices. The MIMIC-IV end point is short-term and postacute; eGFR change may reflect transient acute kidney injury, fluid status, sepsis, or exposure to nephrotoxins. Death competes with renal follow-up, and laboratory ascertainment among survivors can induce selection; fixed-horizon competing outcomes and inverse-probability weighting reduce but do not eliminate these concerns. MIMIC-IV categories were based on eGFR alone and do not represent complete KDIGO G-A risk strata; Greek proteinuria adjustment likewise does not constitute formal 2D staging. The unequal cohort sizes and different NLP pipelines preclude formal model validation, measurement-invariance testing, or direct numerical comparison of effect sizes. The narrow CIs around stage-specific AUCs, only slightly above 0.50, indicate that statistical precision should not be equated with clinically meaningful discrimination; residual within-category differences in continuous eGFR, acute illness, and documentation intensity may still contribute to the observed signal. The Greek mixed model used 314 independent patient clusters for 10 nonintercept fixed-effect terms and converged; the AIC favored the NLP terms, whereas the BIC did not. Stability was supported by closely similar binary associations for the original score (OR=1.49, 95% CI 1.04‐2.14), patient-level held-out TF-IDF score (OR=1.48, 95% CI 1.03‐2.12), and expanded 287-patient analysis (OR=1.39, 95% CI 1.08‐1.79), but these checks do not validate individual prediction or subgroup-specific coefficients. Clinical-group coefficients were adjustment terms only. Neither pipeline explicitly modeled negation scope; the Greek pipeline did not systematically harmonize Greeklish, and neither content-validation procedure used a second annotator or retained a blinded passage-level test set.

### Clinical Implications

The overlaid decision-curve analysis distinguishes cross-setting replication of association from incremental clinical utility. In MIMIC-IV, adding maximum polarity and negativity to the clinical model produced an incremental net benefit close to 0 across thresholds (maximum approximately 0.0015), indicating limited additional decision value for fixed-horizon classification beyond the measured clinical variables. In the separate Greek CKD cohort, adding the clinician-validated TF-IDF negativity score produced larger but unstable changes in net benefit, including positive values at thresholds of 0.30 and 0.50 and a negative value at 0.35. The magnitude and direction of incremental utility therefore depended on the cohort, clinical setting, feature construction, end point, and modeling specification. Because these factors differed simultaneously, the contrast cannot be attributed specifically to language or sample size. Importantly, these decision-analytic findings do not negate the replicated associations with renal deterioration; rather, they demonstrate that clinically relevant information is present in narratives across settings, while its incremental value for individual decision-making remains context dependent and requires setting-specific validation.

### Future Directions

Future work should prospectively evaluate transportability using a common outcome definition and a harmonized text representation, retain patient-level separation during feature fitting, assess explicit negation and temporality, and perform blinded multirater annotation. Clinical utility should be evaluated only after external calibration and decision-analytic benefit are demonstrated.

## Supplementary material

10.2196/94249 Multimedia Appendix 1 Supplementary figures (cohort flow, sensitivity analyses, competing-outcome text-feature associations, and model calibration), extended methodology for natural language processing and statistical analyses, cohort summaries, and data governance details.

## Abbreviations

AIC: Akaike information criterion
AKD: acute kidney disease in the context of chronic kidney disease
AUC: area under the curve
BIC: Bayesian information criterion
CKD: chronic kidney disease
CKD-EPI: Chronic Kidney Disease Epidemiology Collaboration
eGFR: estimated glomerular filtration rate
EHR: electronic health record
HD: hemodialysis
KDIGO: Kidney Disease: Improving Global Outcomes
MIMIC-IV: Medical Information Mart for Intensive Care IV
NLP: natural language processing
NTx: kidney-transplant follow-up
OR: odds ratio
RMSE: root mean square error
RRR: relative risk ratio
TF-IDF: term frequency–inverse document frequency
