# Supplementary material for: Context-Dependent Clinical Narrative Signals of Renal Function Deterioration in Cross-Language Settings: Retrospective Multicohort Study
Source: JMIR Med Inform. 2026 Nov 2;14:e94249. doi: 10.2196/94249 (PMC13727024; doi:10.2196/94249)
Supplement: Supplementary file 1 [file medinform-v14-e94249-s001.docx]

Supplementary Material

# Contents

- Figure S1 Cohort flow and model-specific analytical denominators
- Figure S2 Greek score-stability and outcome-association sensitivity analyses
- Figure S3 Primary MIMIC-IV fixed-horizon competing-outcome text-feature associations.
- Figure S4 Five-fold out-of-fold calibration of the primary MIMIC-IV models.
- S1. Study design, cohorts, and analytic roles
- S2. Renal measures, outcome definitions, and KDIGO simplification
- S3. Clinical-text preprocessing and feature construction
- S4. Annotation-based validation, lexicons, anchors, and synthetic examples
- S5. Statistical models, complexity diagnostics, and sensitivity analyses
- S6. MIMIC-IV cohort flow, competing outcomes, calibration, and dependence diagnostics
- S7. Summary of all sub-cohorts used in the statistical analyses
- S8. Software, reproducibility, data governance, and intellectual-property status
- S9. Ethics and data protection
- Supplementary references

**Figure S1.** Cohort flow and model-specific analytical denominators. Numbers refer to admissions until the MIMIC-IV index-admission rule and to patients thereafter. The first eligible live-discharge admission was selected per patient. MIMIC-IV denominators vary by mortality, renal ascertainment, and competing-outcome requirements. Greek denominators vary by repeated eGFR measurements, trajectory classification, TF-IDF availability, and clinical covariates.

**Figure S2.** Greek score-stability and outcome-association sensitivity analyses. The forest plot compares original, repeated held-out inverse-document-frequency (IDF), worst-case IDF-decontaminated, clinically adjusted, proteinuria-adjusted, and expanded-overlap specifications. Points are odds ratios for renal deterioration per 1-SD higher adverse-language score, and horizontal bars are 95% confidence intervals. The dashed vertical line denotes an odds ratio of 1.00. Cohort-specific analytic sample sizes are printed in the row labels.

**Figure S3.** Primary MIMIC-IV fixed-horizon competing-outcome text-feature associations. The model included 87,533 patients: 57,813 alive without recorded deterioration, 24,821 with renal deterioration, and 4,899 deaths by 90 days. Relative risk ratios compare renal deterioration or death with survival without recorded deterioration and are per 1-SD higher text feature. Both content measures were included simultaneously with age, sex, admission type, discharge eGFR G category, prior NLP-scored admissions, and documentation volume. Points are adjusted relative risk ratios and horizontal bars are model-based 95% confidence intervals. The dashed vertical line denotes 1.00.

**Figure S4.** Five-fold out-of-fold calibration of the primary MIMIC-IV models. The analysis included 87,533 first eligible live-discharge patients. Separate panels show one-versus-rest calibration for renal deterioration and death by 90 days. Within each model and outcome, predictions were divided into 10 equal-frequency bins. Points plot the observed event proportion against the mean predicted probability; vertical bars are Wilson 95% confidence intervals for the observed binomial proportion. Lines connect bins for visual guidance, and the diagonal dashed line indicates perfect calibration. The clinical-plus-NLP model added adverse-language similarity and negative-versus-reassuring polarity to the clinical base model.

# S1. Study design, cohorts, and analytic roles

This retrospective multi-cohort study analyzed electronic health record narratives and renal laboratory data from Heraklion University Hospital in Crete, Greece, and MIMIC-IV [1]. The cohorts were analyzed independently. MIMIC-IV served as a cross-language and cross-setting replication cohort, not as formal external validation of the Greek TF-IDF model, because the populations, text representations, endpoints, and statistical models differed.

## Greek source cohort

The Greek clinical-text source cohort included 314 adults with at least two recorded renal-function measurements and at least 90 days of observation: 170 with nondialysis chronic kidney disease (CKD), 103 receiving maintenance hemodialysis (HD), 22 with acute kidney disease in the context of CKD (AKD), and 19 undergoing kidney-transplant follow-up (NTx). No peritoneal-dialysis patients were included.

The groups had different clinical meanings but entered the primary mixed model as adjustment categories. Dialysis was represented as the lowest renal-function reference state. Recorded HD eGFR was not interpreted as a native residual-kidney trajectory or as a deterioration endpoint. The higher descriptive eGFR in NTx reflects expected functioning-graft status and was not interpreted as a treatment effect or study finding. Maintenance-HD narratives also served as an outcome-independent reference corpus during Greek lexicon development and TF-IDF normalization; HD was never labeled as a decreasing-eGFR outcome class.

| **Characteristic** | **Maintenance-HD patients (n=103)** |
| --- | --- |
| **Female gender, n (%)** | 49 (47.6) |
| **Male gender, n (%)** | 54 (52.4) |
| **Age, years, mean (SD)** | 66.8 (16.0) |
| **Elapsed time since baseline at recorded measurements (range)** | 519 (0–1730) |
| **Recorded eGFR, mL/min/1.73 m², mean (SD)** | 13.9 (3.6) |
| **Baseline proteinuria, mg/g creatinine, mean (SD)** | 2150.1 (5276.4) |

**Table S1.** Characteristics of the maintenance-hemodialysis reference group. Values characterize source records only. In the mixed model, HD was the patient-group reference state; these measurements were not interpreted as native residual-kidney trajectories. Elapsed time was calculated for each measurement relative to the patient’s first recorded measurement, assigned day 0. The reported range therefore includes baseline observations. These values describe measurement timing, not the distribution of patients’ total follow-up durations

## MIMIC-IV source and patient-level analysis cohorts

The text source comprised 331,793 NLP-scored admissions from 145,914 patients. Patient-level outcome analyses used the first eligible live-discharge admission to avoid overlapping follow-up and repeated-patient dependence. Complete-case denominators differed by endpoint: 112,653 for 90-day mortality, 82,634 survivors for the renal-deterioration logistic model, and 87,533 for the primary fixed-horizon competing-outcome model.

# S2. Renal measures, outcome definitions, and KDIGO simplification

Serum creatinine was converted to eGFR using the CKD-EPI 2021 equation [2]. Greek follow-up extended to a maximum of 1,797 days. Baseline proteinuria was log-transformed and included as a continuous covariate in the Greek model.

In MIMIC-IV, baseline eGFR was the median outpatient value 365 to 7 days before admission. Derived features included inpatient nadir and discharge eGFR. The primary 90-day analysis classified patients as alive without recorded renal deterioration, alive with renal deterioration, or dead, with death prioritized if both death and renal deterioration were recorded. Renal deterioration was determined using the prespecified numerical and stored trajectory criteria in the source pipeline.

**KDIGO simplification.** KDIGO classifies CKD using both GFR (G) and albuminuria (A) categories and requires evidence of chronicity [3]. Albuminuria and chronicity were not consistently available in MIMIC-IV; therefore, G1–G5 are eGFR G categories only and are not complete KDIGO G–A risk strata. In the Greek cohort, proteinuria adjustment was available but did not uniformly support A1–A3 classification. Both analyses should therefore be interpreted as renal function analyses with separate adjustment for proteinuria, where available, rather than as complete KDIGO staging.

# S3. Clinical-text preprocessing and feature construction

## Greek TF-IDF pipeline

Greek narrative fields were converted to lowercase, Unicode-normalized, stripped of diacritics and punctuation, tokenized, and lemmatized. Greek stopwords and nonalphabetic tokens were removed, and low-frequency terms were excluded. Patient-level TF-IDF vectors were L2 normalized. The final Greek adverse-language score was constructed solely from clinician-validated TF-IDF entries; the 384-dimensional history embeddings retained in intermediate files were auxiliary and not included in the final score.

For patient i, the adverse-language score was Scoreᵢ = Σₜ∈W validated TFIDFᵢₜ, where W validated is the set of 21 category-term entries classified as worsening/adverse. The score was finalized without using serial eGFR trajectories or deterioration labels. Text features and independently constructed renal outcomes were linked only for downstream association analyses.

## English embedding pipeline

MIMIC-IV discharge summaries were normalized for line endings, divided at paragraph boundaries, and combined or split into contextual chunks of approximately 1,000 characters (operational range approximately 400–1,400). Long paragraphs were split near sentence boundaries and empty chunks were removed. No word-level sentiment dictionary, stemming, or lemmatization was applied.

Chunk and anchor embeddings were generated with sentence-transformers/all-MiniLM-L6-v2, batch size 64, and L2 normalization [4]. For normalized chunk embedding eᵢ, adverse anchor embeddings sⱼ⁻, and reassuring anchor embeddings sₖ⁺: Nᵢ=(1/27)Σⱼ cos(eᵢ,sⱼ⁻); Rᵢ=(1/8)Σₖ cos(eᵢ,sₖ⁺); and Pᵢ=Nᵢ−Rᵢ. Nᵢ is adverse-language similarity (negativity), Rᵢ reassuring-language similarity, and Pᵢ the negative-versus-reassuring semantic contrast (polarity). Admission-level maximum values were primary, and 95th-percentile values were sensitivity features. The three highest-scoring adverse passages per processed admission were exported for qualitative review. Documentation volume was the log-transformed chunk count and was treated as a utilization/acuity covariate rather than a content feature.

# S4. Annotation-based validation, lexicons, anchors, and synthetic examples

## Greek clinician-led term-in-context review

A native Greek-speaking nephrologist reviewed all 86 candidate category-term entries together with concordance passages from the source corpus. Each entry was classified as worsening/adverse, improvement/reassuring, stable, or irrelevant/ambiguous/treatment-related/anatomical. Thirteen entries (15.1%) were rejected, leaving 73 validated category-term entries; 21 entries initially classified as worsening/adverse formed the adverse-language score used in the reported analyses. During subsequent review, six of these worsening/adverse entries (ασυμπιεστη, πιεσεις, πιεσεων, πιεσεως, πιεση, πιεσης) and one improvement/reassuring entry (οξυγονοθεραπεια) were reclassified and were therefore not included in the corresponding final subsets presented in the following supplementary tables. This subsequent reclassification affected the presentation and clinical categorization of the lexicon but did not alter the 21-entry negativity score used in the reported analyses. This was content and face validation of clinical-status language rather than outcome-driven term selection or psychometric validation of emotional sentiment.

| **Clinical-status category** | **No. of retained entries** | **Retained Greek category-term entries** |
| --- | --- | --- |
| **Worsening/adverse** | 15 | ανησυχια, ανησυχιας, δυσκολια, δυσκολιας, εξανθημα, εξανθηματα, εξανθηματος, επιβαρυνση, επιδεινωθηκε, επιδεινωμενη, επιδεινωμενης, επιδεινωνεται, επιδεινωση, επιδεινωσης, επιπλεγ |
| **Improvement/reassuring** | 31 | ακτινοθεραπεια, αναπληρωση, αναστροφα, αναστροφη, ανταποκριθηκε, ανταποκριση, ανταποκρισης, αποθεραπεια, αποκατασταθηκε, αποκατασταση, αποκαταστασης, βελτιωθει, βελτιωθηκαν, βελτιωθηκε, βελτιωμενη, βελτιωμενος, βελτιωση, βελτιωσης, διορθωμενο, διορθωση, θεραπεια, θεραπειας, θεραπευτικα, θεραπευτικη, θεραπευτικης, θεραπευτικο, θεραπευτικου, κορτιζονοθεραπεια, κορτιζονοθεραπειας, υφεση, υφεσης |
| **Stable** | 20 | αμεταβλητη, αποκατασταθηκε, ασταθεια, ασταθειας, ασταθης, ισορροπιας, ομαλα, ομαλη, ομαλο, ομαλοποιηση, σταθερα, σταθερη, σταθερης, σταθερο, σταθεροποιηθηκε, σταθεροποιηση, σταθεροποιησης, σταθερος, σταθερου, συσταθει |

**Table S2.** Complete revised Greek clinical-status lexicon (66 category-term entries after reclassification). The same normalized token may appear in more than one provisional clinical-status category when contextual use supported more than one assignment.

| **Excluded entry** | **Initial category** | **Clinical reason for exclusion** |
| --- | --- | --- |
| **αποκλιμακωση** | Worsening | Therapeutic de-escalation does not itself indicate deterioration. |
| **ασυμπιεστη*** | Worsening | Descriptor of non-compressibility. |
| **ευπιεστη** | Worsening | Descriptor of compressibility. |
| **πιεσεις*** | Worsening | Pressure-related term without an inherent direction of clinical change. |
| **πιεσεων*** | Worsening | Pressure-related term; the same exclusion applies across inflected forms. |
| **πιεσεως*** | Worsening | Pressure-related term; the same exclusion applies across inflected forms. |
| **πιεση*** | Worsening | Pressure-related term; the same exclusion applies across inflected forms. |
| **πιεσης*** | Worsening | Pressure-related term; the same exclusion applies across inflected forms. |
| **πιεστικα** | Worsening | Pressure-related or compressive descriptor. |
| **πιεστικη** | Worsening | Pressure-related or compressive descriptor. |
| **συμπιεση** | Worsening | Anatomical compression descriptor. |
| **συμπιεσης** | Worsening | Anatomical compression descriptor. |
| **χημειοθεραπειας** | Improvement | Treatment name; its presence does not establish improvement or recovery. |
| **χημειοθεραπειες** | Improvement | Treatment name; its presence does not establish improvement or recovery. |
| **ινσουλινοθεραπεια** | Improvement | Treatment name; its presence does not establish improvement or recovery. |
| **οξυγονοθεραπεια*** | Improvement | Treatment name; the same exclusion applies to both oxygen-therapy forms. |
| **οξυγονοθεραπειας** | Improvement | Treatment name; the same exclusion applies to both oxygen-therapy forms. |
| **ορμονοθεραπεια** | Improvement | Treatment name; its presence does not establish improvement or recovery. |
| **σιδηροθεραπεια** | Improvement | Treatment name; its presence does not establish improvement or recovery. |
| **σιδηροθεραπειας** | Improvement | Treatment name; its presence does not establish improvement or recovery. |

**Table S3.** Candidate entries excluded from the final presented lexicon after clinician term-in-context review, including entries subsequently reclassified (marked with asterisk).

## English clinically curated anchor expressions

| **Anchor set** | **No.** | **Complete expressions** |
| --- | --- | --- |
| **Adverse** | 27 | worsening clinical status; deterioration; decompensated; multi-organ failure; septic shock; poor prognosis; guarded prognosis; grave prognosis; end-stage disease; complicated by; postoperative complication; bleeding; hemorrhage; thrombosis; reintubation; re-admission; code blue; cardiac arrest; do-not-resuscitate; DNR order; comfort measures only; hospice; palliative care; noncompliant; refused treatment; left against medical advice; poor adherence |
| **Reassuring** | 8 | improved clinically; stable for discharge; no complications; tolerated well; favorable prognosis; uncomplicated course; symptoms resolved; labs improving |

**Table S4.** Complete MIMIC-IV anchor (seed-expression) sets. Anchors represent clinical deterioration, prognosis, complications, treatment response, disposition, and adherence rather than general emotion.

## English passage review and remaining error mechanisms

Investigators reviewed high-scoring passages in their clinical context and classified them qualitatively as affirmative adverse status, reassuring status, neutral/irrelevant, or negated/historical/hypothetical. Because individual labels were not retained as a formal blinded evaluation dataset, sensitivity, specificity, a confusion matrix, and inter-rater agreement are not reported. Neither pipeline implemented explicit negation-scope or temporality detection. Greeklish/transliterated English was not systematically harmonized. These are recognized sources of residual error.

## Fully synthetic examples

| **Language** | **Fully synthetic narrative fragment** | **Illustrative interpretation** |
| --- | --- | --- |
| **Greek** | Η νεφρική λειτουργία παρουσιάζει επιδείνωση και απαιτείται στενή παρακολούθηση. | Contains a retained worsening expression; expected to increase the Greek adverse-language score, depending on corpus TF-IDF weights. |
| **Greek** | Η κλινική εικόνα παραμένει σταθερή και οι εργαστηριακές τιμές βελτιώθηκαν. | Contains stable/reassuring language; does not enter the 21-term adverse sum. |
| **English** | Renal function worsened during an otherwise complicated course. | Expected to have greater similarity to adverse anchors. |
| **English** | Symptoms resolved, laboratory values are improving, and the patient is stable for discharge. | Expected to have greater similarity to reassuring anchors and a lower adverse-minus-reassuring contrast. |
| **English** | There was no evidence of deterioration. | Illustrates a negation-scope risk: the contextual embedding may not always distinguish negated from affirmative deterioration. |

**Table S5.** Synthetic examples created for reproducibility documentation. They contain no patient information and are not verbatim clinical text.

# S5. Statistical models, complexity diagnostics, and sensitivity analyses

## Primary Greek longitudinal mixed model

The primary Greek model included 314 patient clusters and 10,306 repeated eGFR measurements. Time, baseline adverse-language score, and baseline proteinuria were standardized. The model included patient-specific random intercepts and random time slopes. Fixed effects were time, adverse-language score, time-by-score interaction, age, sex, baseline eGFR, log-transformed baseline proteinuria, and clinical group. Dialysis was the lowest renal-function reference state. Patient-group coefficients were not outcomes and were not interpreted as novel findings; the target parameters were the main effect of adverse language score and the time interaction.

Model complexity was evaluated at the patient-cluster level. The model contained 10 non-intercept fixed-effect terms for 314 clusters (approximately 31 clusters per term), used clinically prespecified covariates, did not use stepwise selection, and converged. The two NLP terms improved the likelihood and AIC but not the BIC, indicating evidence of association without demonstrating a fully validated predictive model.

| **Statistic** | **Clinical model** | **Clinical + NLP** | **Comparison** |
| --- | --- | --- | --- |
| **Patients** | 314 | 314 | — |
| **eGFR measurements** | 10,306 | 10,306 | — |
| **Estimated parameters** | 19 | 21 | +2 |
| **Log-likelihood** | −47,745.959 | −47,739.380 | +6.580 |
| **AIC** | 95,529.919 | 95,520.759 | −9.159 |
| **BIC** | 95,667.488 | 95,672.810 | +5.322 |
| **Likelihood-ratio test** | — | — | χ²=13.159; 2 df; P=.0014 |
| **Residual variance** | 574.448 | 574.458 | +0.010 |
| **Random-intercept variance** | 186.127 | 177.510 | −8.617 |
| **Random-slope variance** | 26.182 | 24.577 | −1.605 |
| **Intercept–slope covariance** | 21.319 | 17.310 | −4.009 |
| **Centered-time variance-partition coefficient** | 0.245 | 0.236 | −0.009 |

**Table S6.** Greek mixed-model specification comparison.

## Held-out TF-IDF and binary-outcome sensitivity analyses

To reduce the possibility that corpus-wide TF-IDF estimation encoded outcome information, patient-level fivefold held-out TF-IDF fitting was repeated 50 times. Vocabulary and inverse-document-frequency values were fitted in training patients and frozen before application to held-out patients. Original and held-out scores were nearly identical (Pearson r=0.997; Spearman ρ=0.998). The direction and magnitude of the renal-deterioration associations were stable across specifications.

| **Specification** | **Patients** | **Association with renal deterioration** | **Discrimination** |
| --- | --- | --- | --- |
| **Original Greek score** | 139 | OR=1.49 (95% CI 1.04–2.14); P=.030 | AUC=0.596 (95% bootstrap CI 0.503–0.688) |
| **Patient-level held-out TF-IDF score** | 139 | OR=1.48 (95% CI 1.03–2.12); P=.034 | AUC=0.595 (95% bootstrap CI 0.501–0.685) |
| **Conservatively decontaminated score** | 139 | OR=1.64 (95% CI 1.12–2.41) | AUC=0.618 (95% bootstrap CI 0.523–0.707) |
| **Expanded analysis** | 287 | OR=1.39 (95% CI 1.08–1.79); P=.012 | AUC=0.570 (95% bootstrap CI 0.502–0.635) |

**Table S7.** Greek score-stability analyses. These are association and discrimination sensitivities, not subgroup-prediction models.

## Separate renal-biopsy spline analysis

A distinct Greek renal-biopsy source cohort included 129 patients and 6,072 raw observations; one complete-case model included 106 patients and 5,118 observations. Cubic B-spline models were restricted to 365 days. High versus low adverse-language score was associated with a time-averaged predicted eGFR difference of −4.41 mL/min/1.73 m² over 365 days (95% CI −8.68 to −0.25). This trajectory-area contrast is not a receiver operating characteristic AUC and is not represented in Figure 1.

## Separate 290-patient Greek risk-model sensitivity analysis

| **Metric** | **Standard clinical model** | **NLP-enhanced model** | **Difference/bootstrap result** |
| --- | --- | --- | --- |
| **Patients** | 290 | 290 | — |
| **Renal-deterioration events** | 136 | 136 | — |
| **AUC, point estimate** | 0.724 | 0.714 | −0.010 |
| **Bootstrap mean AUC (95% CI)** | 0.724 (0.666–0.779) | 0.713 (0.658–0.767) | −0.0109 (−0.0437 to 0.0210) |
| **Average precision** | 0.719 | 0.716 | −0.002 |
| **Integrated discrimination improvement** | — | — | 0.0182 |
| **Continuous NRI, point estimate** | — | — | 0.218 |
| **Continuous NRI, bootstrap (95% CI)** | — | — | 0.210 (−0.012 to 0.427) |

**Table S8.** Greek fixed-horizon risk-model sensitivity. The patient-level bootstrap AUC difference (NLP-enhanced minus standard clinical model) was −0.0109 (95% CI −0.0437 to 0.0210). Threshold-specific decision-curve net-benefit differences are reported in Supplementary Table S14.

## Multiplicity and interpretation

The Greek 314-patient longitudinal mixed model and the MIMIC-IV 87,533-patient fixed-horizon competing-outcome model were the primary analyses for their respective cohorts. Alternative aggregation, inverse-probability weighting, stage-specific discrimination, held-out TF-IDF, binary outcome, spline, and decision-curve analyses were sensitivity or secondary analyses. No formal family-wise multiplicity correction was applied; secondary findings were interpreted using effect sizes, confidence intervals, consistency, and clinical plausibility rather than P values alone. For stage-specific discrimination, the supplied admission-level 90-day renal deterioration composite was evaluated against the maximum polarity score across G1–G5 categories. Categories were defined separately from discharge eGFR and from the worst observed eGFR across the baseline-year median, inpatient nadir, and discharge. AUC confidence intervals were estimated using 1,000 nonparametric bootstrap resamples within each category, with subject_id as the cluster so that all admissions belonging to a sampled patient received the same resampling multiplicity.

# S6. MIMIC-IV cohort flow, competing outcomes, calibration, and dependence diagnostics

| **Cohort-flow step** | **Admissions** | **Patients** |
| --- | --- | --- |
| **NLP-scored records** | 331,793 | 145,914 |
| **NLP-scored records with dates** | 331,732 | 145,872 |
| **Live-discharge scored admissions** | 323,656 | 142,103 |
| **First live-discharge scored admission per patient** | 142,060 | 142,060 |
| **Index admissions with discharge eGFR G category** | 112,653 | 112,653 |
| **Survivors with observed renal endpoint before full covariate restriction** | 92,687 | 92,687 |
| **Index admissions with post-discharge death by 90 days** | 5,749 | 5,749 |
| **Known death-prioritized competing outcome before full covariate restriction** | 98,436 | 98,436 |
| **Complete-case renal-deterioration logistic model** | 82,634 | 82,634 |
| **Complete-case fixed-horizon competing-outcome model** | 87,533 | 87,533 |

**Table S9.** MIMIC-IV cohort flow. The complete-case competing-outcome model included 57,813 alive without recorded deterioration, 24,821 with renal deterioration, and 4,899 deaths by 90 days.

## Primary adjusted effects and sensitivity analyses

| **Predictor, per 1 SD** | **Renal deterioration vs no deterioration, RRR (95% CI)** | **Death vs no deterioration, RRR (95% CI)** |
| --- | --- | --- |
| **Polarity** | 1.090 (1.070–1.110) | 1.021 (0.982–1.061) |
| **Negativity** | 0.973 (0.956–0.991) | 1.216 (1.173–1.261) |
| **Documentation volume** | 0.988 (0.970–1.006) | 1.418 (1.367–1.472) |
| **Age** | 0.775 (0.761–0.791) | 2.550 (2.426–2.681) |

**Table S10.** Primary adjusted MIMIC-IV fixed-horizon competing-outcome model (N=87,533; reference outcome, alive without recorded renal deterioration). Maximum feature aggregation was primary. The 95th-percentile polarity sensitivity yielded renal RRR=1.118 (95% CI 1.097–1.139); inverse-probability weighting for renal ascertainment yielded RRR=1.074 (95% CI 1.053–1.095).

## Correlation, variance inflation, and conditional suppression

| **Feature aggregation** | **Correlation sample** | **Pearson r** | **Spearman ρ** | **Polarity VIF** | **Adverse-similarity VIF** |
| --- | --- | --- | --- | --- | --- |
| **Maximum** | 331,793 admissions | 0.468 | 0.450 | 1.384–1.389 | 1.309–1.315 |
| **95th percentile** | 246,829 admissions | 0.489 | 0.465 | 1.418–1.428 | 1.316–1.328 |

**Table S11.** Dependence diagnostics. Correlations were moderate, and VIFs were below 1.5. The weakly inverse adverse-language-similarity coefficient in the joint renal model is interpreted as conditional suppression due to shared construction and covariate adjustment, rather than as a stable inverse or renoprotective association.

## Calibration and incremental predictive performance

| **Outcome** | **Observed / predicted rate** | **AUC (95% CI)** | **Calibration intercept (95% CI)** | **Calibration slope (95% CI)** | **Brier / ECE** |
| --- | --- | --- | --- | --- | --- |
| **No deterioration** | 0.66047 / 0.66048 | 0.6113 (0.6074–0.6152) | 0.020 (−0.007 to 0.046) | 0.969 (0.934–1.004) | 0.21656 / 0.00767 |
| **Renal deterioration** | 0.28356 / 0.28355 | 0.6134 (0.6092–0.6175) | 0.004 (−0.033 to 0.041) | 1.005 (0.967–1.042) | 0.19655 / 0.00868 |
| **Death within 90 days** | 0.05597 / 0.05597 | 0.7749 (0.7688–0.7811) | −0.023 (−0.105 to 0.060) | 0.990 (0.958–1.023) | 0.04958 / 0.00088 |

**Table S12.** One-vs-rest discrimination and calibration for the primary clinical-plus-NLP competing-outcome model. ECE indicates expected calibration error.

| **Incremental metric, NLP model minus clinical base** | **Difference** | **95% CI** |
| --- | --- | --- |
| **Multiclass log loss** | −0.00120 | −0.00154 to −0.00085 |
| **Multiclass Brier score** | −0.00056 | −0.00074 to −0.00037 |
| **One-vs-rest AUC: no deterioration** | +0.00193 | +0.00114 to +0.00273 |
| **One-vs-rest AUC: renal deterioration** | +0.00178 | +0.00103 to +0.00253 |
| **One-vs-rest AUC: death** | +0.00320 | +0.00194 to +0.00446 |

**Table S13.** Incremental predictive performance. Despite statistical precision, the changes are too small to establish clinical utility.

## Additional numerical sensitivity details

| **Threshold probability** | **Incremental net benefit, NLP-enhanced minus standard** |
| --- | --- |
| 0.05 | 0.0000 |
| 0.10 | 0.0000 |
| 0.15 | 0.0000 |
| 0.20 | 0.0009 |
| 0.25 | 0.0023 |
| 0.30 | 0.0212 |
| 0.35 | −0.0098 |
| 0.40 | 0.0034 |
| 0.45 | 0.0050 |
| 0.50 | 0.0172 |

**Table S14.** Threshold-specific decision-curve net-benefit differences in the separate Greek CKD risk-model sensitivity cohort (N=290; 136 renal-deterioration events). Positive values favor the NLP-enhanced model. No confidence intervals were retained for the threshold-specific net-benefit differences.

| **Model specification** | **Text feature, per 1 SD** | **Renal deterioration, OR (95% CI)** |
| --- | --- | --- |
| Feature-specific (primary interpretation) | Polarity | 1.080 (1.061–1.099) |
| Feature-specific (primary interpretation) | Negativity | 1.005 (0.989–1.021) |
| Joint (secondary conditional model) | Negative-versus-reassuring (polarity) | 1.092 (1.071–1.112) |
| Joint (secondary conditional model) | Adverse-language similarity (negativity) | 0.973 (0.956–0.991) |

**Table S15.** Individually adjusted and joint patient-level MIMIC-IV renal-deterioration models among 82,634 90-day survivors with renal ascertainment. All models adjusted for the prespecified clinical and documentation covariates. Feature-specific models provide the primary clinically interpretable estimates; the joint model is a secondary conditional decomposition of correlated text measures.

| **Stage basis** | **G category** | **Admissions** | **Patients** | **Events** | **AUC** | **Patient-clustered 95% CI** |
| --- | --- | --- | --- | --- | --- | --- |
| Discharge eGFR | G1 | 121,288 | 69,284 | 23,815 | 0.518 | 0.514–0.523 |
| Discharge eGFR | G2 | 78,875 | 47,170 | 25,693 | 0.506 | 0.502–0.511 |
| Discharge eGFR | G3a | 28,129 | 17,372 | 10,297 | 0.522 | 0.516–0.529 |
| Discharge eGFR | G3b | 19,986 | 11,737 | 8,006 | 0.529 | 0.521–0.537 |
| Discharge eGFR | G4 | 13,188 | 7,353 | 5,530 | 0.552 | 0.541–0.563 |
| Discharge eGFR | G5 | 13,244 | 4,871 | 3,444 | 0.534 | 0.521–0.547 |
| Worst observed eGFR | G1 | 105,133 | 60,442 | 18,257 | 0.522 | 0.517–0.527 |
| Worst observed eGFR | G2 | 97,678 | 56,949 | 26,645 | 0.518 | 0.514–0.523 |
| Worst observed eGFR | G3a | 38,741 | 23,932 | 12,087 | 0.520 | 0.514–0.526 |
| Worst observed eGFR | G3b | 31,776 | 19,086 | 10,410 | 0.524 | 0.517–0.531 |
| Worst observed eGFR | G4 | 21,880 | 13,357 | 7,713 | 0.534 | 0.526–0.543 |
| Worst observed eGFR | G5 | 27,567 | 13,776 | 8,019 | 0.530 | 0.522–0.538 |

**Table S16.** Stage-specific admission-level discrimination of the maximum polarity score for the supplied 90-day renal-deterioration composite. The analysis includes G5, representing the lowest eGFR category under the supplied data handling. Confidence intervals are percentile 95% intervals from 1,000 patient-cluster bootstrap resamples. The consistently above-chance but small AUCs indicate a statistically detectable within-category narrative signal without clinically useful stand-alone discrimination.

**S7. Summary of all sub-cohorts used in the statistical analyses**

The following table summarizes all sub-cohorts used in the statistical analyses of this paper

**Table S17.** Summary of all sub-cohorts used in statistical analyses

| **Analysis population** | **N** | **Events** | **Inclusion rule** | **Overlap** |
| --- | --- | --- | --- | --- |
| Original longitudinal | 314 | Not applicable | Linked negativity and clinical metadata; 10,306 records. | All Greek analysis populations are contained within the 314-patient source cohort. |
| Structured binary | 139 | 65 of 139 | Structured-concept/embedding export linked to deteriorating/non-deteriorating trajectory labels. | 112 overlaps with the 287-patient populations; contains all 129 biopsy patients. |
| Expanded binary | 287 | 152 of 287 | Original score cohort with an available trajectory label; | Entirely within the 314-patient population; overlaps 112 structured-binary, 107 biopsy, and 88 proteinuria-complete biopsy patients. |
| Exploratory trajectory-derived score | 290 | 136 of 290 | Archived rule: ≥3 eGFR observations and ≥0.5 years of follow-up. | Derived exclusively from the 314-patient population. |
| Biopsy B-spline | 129 | Not applicable | Biopsy-linked longitudinal records available within the structured export. | Entirely within the 139-patient population; |
| Proteinuria-complete biopsy B-spline | 106 | Not applicable | Biopsy patients with recorded baseline proteinuria. | Entirely within the 129-patient population; |

# S8. Software, reproducibility, data governance, and intellectual-property status

Analyses were conducted in JupyterLab 7.3.2 using Python and standard scientific/statistical packages, including pandas, patsy, scikit-learn, sentence-transformers, and statsmodels. The complete anchor (seed-expression) sets, validated Greek lexicon, excluded candidates, scoring equations, model specifications, denominators, and fully synthetic examples are reported in this Supplement.

MIMIC-IV and MIMIC-IV-Note were accessed through PhysioNet by a credentialed investigator (Ioannis Petrakis) after completion of approved CITI “Data or Specimens Only Research” training and acceptance of the applicable PhysioNet data-use agreement. MIMIC-IV data, notes, and patient-level derived data cannot be redistributed by the authors. Qualified researchers may request independent access through PhysioNet credentialing and acceptance of the applicable agreement. Greek patient-level data require institutional ethics and data-governance approval.

Components of the executable implementation are undergoing institutional patent and technology-transfer review. The complete pipeline therefore cannot yet be deposited publicly. After completion of that process and institutional approval, the authors intend to release non–patient-level analysis code and supporting documentation in a version-controlled repository. Patient-level and row-level MIMIC-derived material will remain governed by the PhysioNet data-use agreement and will not be placed in a public repository.

# S9. Ethics and data protection

All data were de-identified and analyzed retrospectively with no patient contact. The Greek protocol, including use of electronic health record text and laboratory data, was approved by the institutional ethics committee (Approval No. 230/07.03.2025). The requirement for informed consent was waived for anonymized secondary analysis. Greek data handling complied with EU General Data Protection Regulation 2016/679. MIMIC-IV use followed PhysioNet credentialing and its data-use agreement.

# Supplementary references

1. Johnson AEW, Bulgarelli L, Shen L, et al. MIMIC-IV, a freely accessible electronic health record dataset. Sci Data. 2023;10:1. doi:10.1038/s41597-022-01899-x

2. Inker LA, Eneanya ND, Coresh J, et al. New creatinine- and cystatin C-based equations to estimate GFR without race. N Engl J Med. 2021;385:1737–1749. doi:10.1056/NEJMoa2102953

3. Kidney Disease: Improving Global Outcomes (KDIGO) CKD Work Group. KDIGO 2024 Clinical Practice Guideline for the Evaluation and Management of Chronic Kidney Disease. Kidney Int. 2024;105(4S):S117–S314. doi:10.1016/j.kint.2023.10.018

4. Reimers N, Gurevych I. Sentence-BERT: sentence embeddings using Siamese BERT-networks. EMNLP-IJCNLP. 2019:3982–3992. doi:10.18653/v1/D19-1410

5. Salton G, Buckley C. Term-weighting approaches in automatic text retrieval. Inf Process Manag. 1988;24:513–523. doi:10.1016/0306-4573(88)90021-0
